# Diabetes and its associated factors: A Retrospective cohort analysis of a large database at Indus Hospital Health Network

**DOI:** 10.12669/pjms.40.2(ICON).8948

**Published:** 2024-01

**Authors:** Faridah Amin, Muhammad Imran, Syed Asif Hafeez, Beenish Zehra

**Affiliations:** 1 Faridah Amin, PhD. Director and Professor, Indus College of Family Medicine and Public Health, Indus Hospital & Health Network, Karachi, Pakistan; 2 Muhammad Imran, MPH. Lecturer and Research Scientist, ORIC, Indus Hospital & Health Network, Karachi, Pakistan; 3 Syed Asif Hafeez, MSc. Endocrinologist, Department of Endocrinology, Indus Hospital & Health Network, Karachi, Pakistan; 4 Beenish Zehra, MBBS. Resident, Department of Family Medicine, Indus Hospital & Health Network, Karachi, Pakistan

**Keywords:** Diabetes in Pakistan, Non-communicable diseases in Pakistan, diabetes registry Pakistan, NCD registry Pakistan

## Abstract

**Objectives:**

This is a retrospective cohort analysis of diabetes and its associated factors from Electronic Medical Record (EMR) of 2020-2022 of Indus Hospital Health Network (IHHN), Korangi campus Karachi.

**Methods:**

Retrospective cohort study was conducted at Indus Hospital & Health Network (IHHN), Korangi Karachi. Out-patient records of adult patients of 2020-2022 were extracted from EMR of IHHN in March 2023. Descriptive statistics were presented as median (IQR) and frequency and percentage. Chi-square test determined association of risk-factors with diabetes and Wilcoxon-sign-rank test compared change in HbA1C from baseline.

**Results:**

Data of 460,799 adult patients were extracted and analyzed. Median age of patients was 38.71 (27.87-52) years. Female preponderance was observed in our study. Out of 460,799, HbA1C was seen in 42,638 (9.25%) patients. Among these 29,596 (69.4%) had a HbA1C ≥ 6.5% while 13,042 (30.6%) had a HbA1C in the pre-diabetes range. Significant association was found between age, baseline creatinine, LDL and diabetes with no association depicted between gender, BMI, blood pressure, triglycerides, HDL and diabetes status. Patients in general had higher HbA1C at baseline as compared to last visit (p-value<0.001).

**Conclusion:**

High blood pressure, obesity, increased creatinine, micro albuminurea, high LDL and Triglycerides were important risk factors for diabetes. This study reports a snap shot of the status of diabetes and associated risk-factors in the Pakistani population. This was the first time that a large data was extracted and analyzed from a healthcare institution in Pakistan, which would guide physicians and public health practitioners to take evidence-based decisions for prevention and management of diabetes.

## INTRODUCTION

More than 400 million people have diabetes mellitus worldwide with 1.5 million deaths attributed to diabetes each year with majority from low-middle income countries.^1^ The prevalence of diabetes in South-Asia among adults is estimated to be 33 million in 2021. Around 17% of the Pakistani population has diabetes, ranking Pakistan third in place by International Diabetic Federation (IDF), preceded by China and India. Pakistan had the highest comparative diabetes prevalence rate 30.8%, in 2021 with highest mortality (35.5%) under the age of 60 from diabetes complications. In the provinces of Pakistan, Sindh has the highest diabetes militias prevalence (32.3%), followed by Punjab 30.2%, Baluchistan 29.5% and KPK 13.2%.^2^ Another eleven million adults have impaired glucose tolerance which puts them at higher risk of developing Type-2 diabetes.

A recent survey conducted in Pakistan, found old age (51–60 years, prevalence 26.03%), no formal education (17.66%), class III obesity (35.09%) and family history (31.29%) as key risk factors. Additionally, females exhibited higher prevalence at 17.80% compared to overall prevalence of 10.91%.^3^ In line with global trends, Pakistan has higher prevalence of diabetes in urban areas than rural areas (15.1% vs 1.6%)^4^. For Pakistan, a rapidly urbanizing country in South Asia, it’s expected that half of the country’s population will be living in urban areas by 2025, indicating towards an increasing burden in of NCDs in coming years.^4^

Unfortunately, more than a quarter (26.9%) of Pakistani population with diabetes are un-diagnosed.^5^ The challenge of un-diagnosed diabetes is compounded by low penetration and quality of primary care services in the country, and a less than 1% allotment of GDP to health.^6^ A study conducted in Pakistan reported presence of micro and macrovascular complication in 7% of diabetic patients and uncontrolled diabetes is reported as the main risk factor for all complications.^7^

The direct cost per diabetic patient/annum in Pakistan is an alarming (332 USD); and on average, 19% of earnings of low-income patients are spent on diabetes care.^8^ Yet, there is an absence of cost effective and organized diabetes care services for the poor population from the public platform. The findings of the 2nd National Diabetes Survey of Pakistan, suggests the need for interventions in public and private sector, for controlling diabetes through comprehensive cost-effective diabetes care programs in Pakistan.^9^

Considering this huge gap in service delivery to underprivileged, IHHN began its diabetes management program (DMP) in 2014 at its Korangi campus, Karachi Pakistan. Being a free of cost health care service delivery center, the network serves as a referral point for the underprivileged population of the entire country. Hence thousands of patients have been screened, diagnosed and managed for diabetes and its complications for more than a decade. A non-communicable disease (NCD) registry is being created at an institutional level through EMR system. Presently there are no such registry of diabetes and other NCDs available in Pakistan, and it would be worthwhile to create one and analyze large population based NCD data of Pakistan.

This study is therefore a review of all patients screened, diagnosed and managed for diabetes and prediabetes at IHHN Korangi campus. In addition, we also assess frequency of cardiovascular risk factors (raised blood pressure, dyslipidemia, obesity and raised creatinine among the adult population aged ≥18 years who visited IHHN from 2020-2022. To our knowledge since IHHN has one of the largest patient databases in Pakistan, therefore, outcome of this study would give an insight into the magnitude of diabetes and cardiovascular risk factors in Pakistani population and may begin to form a basis for a national registry of NCDs via IHHN EMR. This would help physicians and public health practitioners to direct further research and Clinical practice to address the rising concern of NCDs, and its complications.

## METHOD

A retrospective cohort study was conducted at Indus Hospital & Health Network, Korangi campus. . We extracted out-patient data (all specialties) of adult patients (≥ 18 years), from the EMR database of IHHN, Korangi campus from 1^st^ January 2020 to 31^st^ December 2022 in March 2023.

Data extracted from EMR department of IHHN included, age, gender, height, weight, systolic blood pressure (SBP), diastolic blood pressure (DBP), and laboratory parameters; glycated hemoglobin (HbA1C), creatinine, lipid profile and urine microalbumin/albumin-creatinine ratio (ACR).

Selected samples were categorized according to HbA1C i.e. ≥ 6.5% were labeled as diabetics and 5.7-6.4% indicated pre-diabetes.^10^ Raised SBP was defined as SBP >140 mmHg and DBP as > 90 mmHg.^11^ A raised creatinine was defined as creatinine level > 1.5 mg/dl.^12^ Obesity was defined as a BMI of ≥ 30 kg/m^2^. According to CDC, patients with low density lipoprotein (LDL) ≥ 100 mg/dl were labeled as raised LDL while triglycerides (TG) level of ≥ 150 mg/dl was considered raised. A high-density lipoprotein (HDL) level of <40mg/dl for men and <50 mg/dl for women was considered “low HDL”.^13^ Microalbuminuria was defined as urine albumin or ACR ≥ 30 mg /day.^14^

### Statistical Analysis

It was performed on SPSS version 26. Descriptive statistics like median and inter-quartile range was reported for quantitative variables such as age, BMI, SBP, DBP, LDL, HDL and triglyceride. While frequency and percentage were reported for categorical variables such as gender, raised blood pressure, HbA1C, creatinine, LDL, TG, microalbuminuria, low HDL and obesity. Kolmogorov Smirnov test was used to assess normality of continuous variable. Chi-square test was used to determine the association of demographic and cardiovascular risk factors with HbA1C categories (diabetes and pre-diabetes). Wilcoxon sign rank test compared the change in HbA1C from baseline. A p-value of ≤ 0.05 was considered statistically significant. As data were retrieved from retrospective records, there were missing data; and we analyzed data available for each variable due to which total number of observations varied for all variables.

### Ethical Exemption

Exemption from ethical approval was received from Institutional Review Board Ref:(IRB#: IHHN_IRB_2022_05_015).

## RESULTS

Data of 460,799 adult patients were extracted and analyzed. Median age of patients was 38.71 (27.87-52) years. Majority of patient in our study was female (54.4%). Moreover, the median BMI of the patient was 23.7 kg/m^2^ with SBP of 120 mmHg and DBP of 78 mmHg. However, the median LDL was 99 mg/dl, HDL 37mg/dl and triglyceride 143 mg/dl.

**Fig.1:**
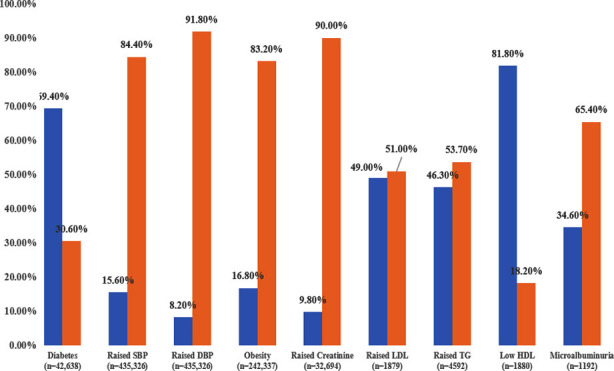
Frequency of diabetes, raised SBP & DBP, obesity, dyslipidemia, microalbuminuria and raised creatinine at baseline among study population (N=460,799)

The raised SBP, DBP, HbA1C, creatinine, LDL, TG, microalbuminuria, low HDL and obesity are shown in Fig-1. Out of 460,799, HbA1C was available for 42,638 (9.25%) patients. Among these 29,596 (69.4%) had a HbA1C ≥ 6.5% while 13,042 (30.6%) had a HbA1C in the pre-diabetes range. SBP and DBP readings were available for 435,326 (94.5%) patients, out of which the SBP was raised among 67,941 (15.6%) whereas DBP was raised among 35,796 (8.2%) of the patients (75,472 (17.3%) has either SBP or DBP raised). Among 241,454 (52.4%) patients with an available BMI, 40,477 (16.8%) patients were obese. Creatinine was available for 32,694 (7.1%) patients, amongst which a raised level was found in 3218 (9.8%). Urinary albumin was available for 1185 (0.3%) patients, out of which 412 (34.8 %) patients had microalbuminuria. Among 460,779, LDL, HDL and triglyceride were available for 1879 (0.4%), 4592 (1.0%) and 1880 (0.4%) patients respectively. Out of these, raised LDL was observed in 921 (49%), raised HDL 1537 (81.8%) and raised triglycerides 2127 (46.3%).

The association of various risk factors associated with HbA1C in diabetic or pre-diabetic range are shown in Table-I. A raised creatinine level was seen in 2300 (10.1%) of diabetic patients. Significant association was found between age, baseline creatinine, LDL and diabetes. Patients age <65 years were significantly higher in diabetes range HbA1C as compared to pre-diabetic (69.9% vs 30.1%, p-value 0.001). Similarly, a raised creatinine was significantly higher among patients with HbA1C in diabetic range as compared to pre-diabetic patients (71.5% vs 28.5%). Furthermore, raised LDL was found higher in patients with diabetes as compared to pre-diabetic patients. However, no association was found between gender, BMI, blood pressure, triglycerides, HDL and diabetes status.

**Table-I:** Association of risk factors with HbA1C levels in diabetes and pre-diabetes range (n=42,638)

Variable	Diabetes Status		p-value

	Pre-Diabetes n (%)	Diabetes n=n (%)	
***Age***			
< 65 Years	10649 (30.1)	24680 (69.9)	<0.001^€^
≥ 65 Years	2393 (32.7)	4916 (67.3)	
***Gender***			
Male	5266 (30.6)	11955 (69.4)	0.863^€^
Female	7776 (30.6)	17641 (69.4)	
***BMI***			
< 30 Kg/m^2^	4691 (32.9)	9583 (67.1)	0.663*^€^
≥ 30 Kg/m^2^	2494 (33.2)	5028 (66.8)	
***Blood Pressure***			
Normal (SBP ≤140 mmHg & ≤ 90 mmHg)	6969 (32.7)	14337 (67.3)	0.564*^€^
Raised (SBP >140 mmHg or DBP >90 mmHg)	2563 (32.4)	5359 (67.6)
***Creatinine at baseline***			
Normal (Cr ≤ 1.5 mg/dl)	9179 (31.1)	20297 (68.9)	<0.001*^€^
Raised (Cr > 1.5 mg/dl)	918 (28.5)	2300 (71.5)	
***Low Density Lipoprotein***			
Normal (LDL < 100 mg/dl)	302 (31.9)	645 (68.1)	0.011^€^
Raised (LDL ≥100mg /dl)	238 (26.5)	661 (73.5)	
***Triglyceride***			
Normal (TG <150 mg/dl)	739 (31.1)	1639 (68.9)	0.437^€^
Raised (TG ≥150 mg/dl)	668 (32.2)	1409 (67.8)	
*High Density Lipoprotein*			
Normal (HDL ≥40mg/dl for men and ≥50 mg/dl for women)	89 (26.6)	245 (73.4)	0.241^€^
Low <40mg/dl for men and <50 mg/dl for women	452 (29.9)	1061 (70.1)	

* Total not equal due to missing data

€ Chi-Square, Total N for age (42,638), gender (42,638), BMI (21,769), Blood Pressure (29,228), creatinine (32,694), LDL (1,846), Triglyceride (4,455) and HDL (1,847).

Our study showed that more patients had higher HbA1C at baseline than in last visits (p-value <0.001). Similarly, there was a significant drop in HbA1C levels at last visit in diabetes patient (p-value <0.001). Whereas, among patients with a pre-diabetes, a significant increase in HbA1C levels was observed at last visit (p-value 0.001) [Table-II].

**Table-II:** Change in HbA1C at follow up among diabetes and pre-diabetes range HbA1C at baseline (n=42,638).

Variable	Baseline	Follow-up	Negative Mean Rank (N)	Positive Mean Rank (N)	p-value
Pre-Diabetes	6 (5.8-6.2)	6 (5.7-6.4)	1021 (1107)	1196 (1110)	0.001*
Diabetes	8.7 (7.4-10.6)	7.8 (6.8-9.4)	4336 (5566)	3305 (2472)	<0.001*
Overall	7.5 (6.2-9.7)	7.3 (6.3-8.9)	5523 (6673)	4390 (3582)	< 0.001*

* p-value <0.05 (Wilcoxon Sign Rank test).

## DISCUSSION

Our study found that, 69.4% were diabetic while 30.6% patients were pre-diabetic. A recent meta-analysis showed a 13.7% pooled prevalence of diabetes in Pakistan with a higher prevalence in urban than rural areas.^15^ The prevalence of pre-diabetes was 11.43% in another meta-analysis in Pakistani population, but a quarter of patients remain un-diagnosed.^16^ It is possible that diabetes was diagnosed by other means such as IGT random or fasting blood sugar levels in patients whose HbA1C levels were not reported.

Around 10% of diabetic patients had raised creatinine, which is consistent with previous studies from Pakistan.^17^ Moreover, SBP or DBP was raised in 15.6% of the patients, indicating the prevalence of uncontrolled or un-diagnosed hypertension in adult population. These findings do not correlate with previous surveys, which reported a prevalence of hypertension as high as 46% among adults in Pakistan.^18^ This discrepancy could be due to the fact that we were unable to identify known hypertensive patients / controlled hypertension from the data. Moreover, an isolated finding of SBP and DBP is inadequate to label patients as hypertensive.

A high prevalence of dyslipidemia among the population is comparable to statistics from recent findings of the National Diabetes survey of Pakistan with majority of Pakistani population having a low HDL.^19^, ^20^ The frequency of a raised creatinine and microalbuminuria was recorded to be 9.8% and 34.6% respectively. In another countrywide analysis, prevalence of nephropathy in all age groups was found to be 21.2% which is not in line with our study.^21^ A relatively higher frequency of microalbuminuria may be explained by the fact that the level was checked amongst high-risk patients only. A 16.8% prevalence of obesity was recorded in this study which is slightly higher than the country figures stated by the World Obesity Federation.^22^

Gender, BMI and raised blood pressure were not associated with diabetes however, as the comparison was with the HbA1C level in pre-diabetic range, we do not expect a significant difference as risk factors are similar for both groups. We found a higher HbA1C level among younger age group as compared to those >65 years of age and this is explained by a higher incidence of diabetes among adults <65 years of age.^23^ However, raised creatinine was more likely with higher HbA1C levels as reported in previous studies.^6^,^24^

Similar to previous studies^19^,^20^, this study showed an association of raised LDL with higher HBA1C level, but there was no association of TG and HDL with HbA1C which contrasts with findings of another study.^25^

The HbA1C improved after follow up in patients who had a higher HbA1C, which is explained by the pharmacological intervention which may have been given to control diabetes. However, a rise in HbA1C level among the pre-diabetic group is concerning as it translates into a lack of compliance to lifestyle modifications. Though there are efforts to increase availability of oral hypoglycemics and insulin to the non-affording patients visiting IHHN, interventions should also be directed towards preventing hyperglycemia among pre-diabetic patients. This would be a more cost-effective intervention as “an ounce of prevention is worth a pound of cure”.

### Limitations

Though this is the first effort to compile data of diabetic patients and those at risk at IHHN, Karachi, we faced some limitations. Management and followed-up of patients with NCDs were limited due to frequent lock-downs during COVID pandemic; and owning to rapid influx of COVID patients, maximum attention was diverted to them. In this period data coding for other diseases was set-aside, consequently creating a back-log of data coding; therefore, data extraction became a challenge. This created a discrepancy in numbers in groups, making it difficult to analyze and compare data to extract meaningful results for deducing plausible inferences. There was no data for normal HbA1C for which it’s hypothesize that HbA1C was requested either in diabetic patient or high-risk patient owning to high cost of its test.

## CONCLUSION

High blood pressure, obesity, increased creatinine, micro albuminurea, high LDL and Triglycerides were important risk factors for diabetes. This is the first study of its kind from one of the largest health care network and database of diabetic patients in Pakistan at the IHHN. The results of this study provide an insight to dig more deeply into the risk factors, cost-effectiveness of interventions and barriers to care for the exploding menace of NCDs. The data extraction and analysis will also guide better EMR management and coding for better utility in extrapolating findings from such a huge database through artificial intelligence.

### Authors` Contribution:

**F.A:** Conception, designed, draft manuscript and final approval of the manuscript to be published.

**MI:** Statistical Analysis, interpretation and result writeup and responsible for the accuracy of the study.

**AH:** Review critically for important intellectual content and final approval of version to be published.

**BZ:** Conception, designed, data acquisition and final approval of version to be published.
